# Measuring benefit from non‐surgical interventions in otolaryngology for different conditions, using the revised 5‐factor Glasgow Benefit Inventory

**DOI:** 10.1111/coa.13992

**Published:** 2022-10-23

**Authors:** Haytham Kubba, William M. Whitmer, George G. Browning

**Affiliations:** ^1^ Hearing Sciences ‐ Scottish Section University of Nottingham, Glasgow Royal Infirmary Glasgow UK

**Keywords:** benign paroxysmal positional vertigo, hearing aids, otolaryngology, outcomes research, tinnitus

## Abstract

**Objectives:**

The Glasgow Benefit Inventory (GBI) has been extensively used to report the benefit from otolaryngological surgery. Benefit from non‐surgical management has not been reported, despite this being the outcome of most otolaryngology and audiology consultations.

**Design:**

GBI responses from 4543 adults from the Scottish ENT Outcome Study were categorised by diagnosis. Benefit scores for different interventions within diagnostic categories for which surgery was not a potential management are reported using the revised 5‐factor Glasgow Benefit Inventory (*GBI‐5F*; 15 questions and 5 factors).

**Setting:**

Adult otolaryngology outpatient clinics in six university hospitals.

**Participants:**

Adults seen with conditions that had no surgical option and given non‐surgical management.

**Results:**

Overall, 80% of participants managed in Scottish Ear Nose and Throat Outcome Study (SENTOS) did not have surgery. A total of 1373 (30%) participants with various diagnoses were given reassurance and advice with no active intervention. There was no change in their *GBI‐5F* total or factor scores, suggesting that they did not come to harm from their lack of active intervention. Hearing aids for bilateral sensorineural hearing loss gave greater benefit than reassurance in all factors, though individuals with a conductive impairment reported greater benefit in the *Quality of life* factor than those with a sensorineural impairment. Hearing aids and maskers produced benefit in the *Support* factor for patients with tinnitus. Epley's manoeuvre for benign paroxysmal positional vertigo gave benefit in the total score and the *Quality of life* factor compared with reassurance. Systemic medication for laryngo‐pharyngeal reflux and topical medication for otitis externa gave no greater benefit in any factor or the total score compared with reassurance.

**Conclusion:**

The *GBI‐5F* and its five factors give useful information for reporting the benefit of non‐surgical interventions in adult otolaryngology and audiology clinics.

**Key points**

- This article is based upon a national audit of the management for their clinical diagnosis of 4543 otorhinolaryngological and audiological adult patients, made at their first outpatient appointment. Only 20% of patients subsequently had surgery. Patients with a diagnosis that did not have a recognised surgical option were studied.
- The 30% of patients managed solely by reassurance showed no major change over the following 6 months. This supports reassurance as a valid management of these patients, as their 5‐factor Glasgow Benefit Inventory scores did not become negative, suggesting that their clinical condition did not deteriorate.
- Provision of a hearing aid for those with a bilateral sensorineural hearing impairment gave significant benefit in the *Quality of life* factor compared with those who only had reassurance. *Quality of life* benefit was greater for those given hearing aids for a conductive loss rather than for a sensorineural loss.
- Patients with tinnitus given a hearing aid or masker reported greater benefit in the *Support* factor compared with those managed by reassurance alone. Patients who had an Epley manoeuvre for benign paroxysmal positional vertigo had greater benefit in the *Quality of life* factor compared with those who received only reassurance.
- Medications for otitis externa and reflux oesophagitis did not lead to any greater reported benefit than reassurance alone.

## 1 INTRODUCTION

Originally described in 1996, the Glasgow Benefit Inventory (GBI) is an 18‐item questionnaire for measuring patient benefit after otorhinolaryngological (ORL) interventions.^1^ Administered after intervention, it measures the change in health status, whether positive (benefit) or negative (harm). It was designed to be patient‐orientated, sensitive to change after intervention and suitable for comparing different interventions. Because it requires no measurement before the intervention, it is easy to use and adaptable to various clinical situations. Since 1996, the GBI has been used on a wide range of ORL surgical operations, with Hendry et al reviewing 117 reports up to January 2015.^2^ We recently described a shorter (15‐question) version of the GBI which we refer to as 5‐factor GBI (*GBI‐5F*).^3^ This has five factor scores which give more detailed information on the specific areas of patient benefit.

SENTOS was a prospective cohort study of patients attending outpatient ORL clinics at six Scottish NHS hospitals between 2001 and 2005.^4^ At that time, all audiological referrals were made via ORL. The study administered two outcome measures: the Health Utilities Index mark 3 (HUI‐3) and the GBI. Only the HUI‐3 results have been reported in detail.^4^ GBI questionnaires were completed by 4543 SENTOS participants 3–6 months after intervention, giving a considerable eset for analysis. This enables study of patient‐reported benefit from a wide range of interventions.

To date, only one paper has reported a non‐surgical intervention (provision of hearing aids).^3^ The article's objective is to report the use of the GBI on a wider range of non‐surgical interventions. Our aim is, firstly, to demonstrate that non‐surgical interventions have measurable patient benefit and, secondly, that the five factors of the new *GBI‐5F* can give useful information on the pattern of patient benefit that is seen in different clinical situations.

## 2 METHODS

The dataset comprised GBI responses obtained from adult patients (16 years or older) attending an NHS Academic ORL outpatient appointment and completing the GBI for the SENTOS study. Details of this cohort have been published previously.^4^ Briefly, 9005 adult patients attending ORL outpatient clinics in one of six Scottish hospitals between 2001 and 2005 were sent the HUI‐3 and GBI questionnaires to complete sometime after the hospital attendance: 6 months later if they underwent surgery or were given hearing aids, 3 months later if they were managed medically or with no active intervention. The HUI‐3 results have already been reported in detail and will not be discussed further here.

The participants completed the original 18‐question GBI, but 3 questions (Q9, Q10 and Q14) were removed to fit the *GBI‐5F* scheme.^3^ The five factors are *Quality of life, Support, Social involvement, Self‐confidence* and *General health*. Each of these, as well as the overall score, are calculated by scoring the responses to each question on a 5‐point scale from −2 to +2, adding up the question scores and then re‐scaling the result from −100 (maximum possible harm) to +100 (maximum possible benefit) and centred on 0 (no change). The total score and factor score each stand alone: they are not sub‐scale scores in the sense that the total score cannot be calculated by adding up the factor scores, for example.

Data were analysed using SPSS version 26 (IBM Corporation, Armonk, New York, USA). Statistical comparisons were made using Kruskal‐Wallis one‐way ANOVA and Mann–Whitney *U*‐test where appropriate. This report referenced to the COSMIN guideline for patient‐reported outcome measures.^5^

## 3 RESULTS

### 3.1 Patient characteristics

Of the 9005 participants in the SENTOS study, 1774 (19.7%) were coded as undergoing surgery. The remaining 7231 (80.3%) did not.

In total, 40 of the questionnaires had been administered to children aged 14 and 15 years, and these were excluded along with five adults undergoing cancer radiotherapy. Of the remaining adults, 4543 of 8960 (51%) completed a GBI questionnaire 6 months after completing treatment. This comprised 1939 men (42.7%) and 2604 women (57.3%), with a median age of 55 years (mean 54 years, range 16–101 years).

Patients with a single clear diagnosis and a single intervention were identified. Those with combined interventions (e.g., ear medication and a hearing aid) were excluded. Patients referred outside ORL/audiology to other departments, including physiotherapy (*n* = 39) and speech therapy (*n* = 109), were excluded. We identified a series of common diagnosis or intervention groups from the dataset for which there was no surgical treatment option and for which there were at least 50 patients for analysis. This gave us the following patient groups: sensorineural hearing loss, conductive hearing loss managed with hearing aids, tinnitus, benign paroxysmal positional vertigo, otitis externa and laryngo‐pharyngeal reflux, plus a large heterogenous group of patients managed by means of reassurance and advice without any active intervention.

### 3.2 No active intervention/reassurance

SENTOS contains a large group of patients coded as receiving ‘reassurance’ or ‘advice on self‐management’ with no active medical or surgical intervention. There were 1373 such patients (30% of those with a completed GBI), 550 men (40.1%) and 823 women with a median age of 55 years (range 16–93, mean 53.77 years). Their primary presenting symptoms included hearing impairment (370 cases, 26.9%), dizziness (217, 15.8%), tinnitus (140, 10.2%), otalgia (110, 8%), hoarseness (94, 6.8%), lump in throat (75, 5.5%) and sore throat (69, 5%). The most common diagnoses then given were ‘no abnormality demonstrated’ (335, 24.4%), ‘bilateral sensorineural hearing loss’ (294, 21.4%), ‘somatoform disease including hyperventilation and globus hystericus/pharyngeus’ (78, 5.7%), “tinnitus” (74, 5.4%), ‘dizziness and light‐headedness’ (50, 3.6%) and vestibular neuronitis (45, 3.3%).

Apart from a very small number of outliers reporting large benefits and harms, most patients report no change in any factor, with 80% scoring zero for *Support*, 65% for *General health*, 60% for *Quality of life*, 80% for *Self‐confidence* and 77% for *Social involvement*. For the total score, 42% score exactly 0 and 62% score between −3.3 and +3.3 (Table 1).

**TABLE 1:** GBI‐5F total and sub‐scale scores for patients (*n* = 1373) with a variety of presenting complaints managed with reassurance and advice only (no active intervention)

Factor	Minimum	First quartile	Median	Third quartile	Maximum
*Support*	−83.3	0	0	0	+100
*General health*	−100	0	0	0	+100
*Quality of life*	−100	0	0	+16.7	+100
*Self confidence*	−100	0	0	0	+100
*Social involvement*	−100	0	0	0	+100
GBI‐5F total score	−66.7	0	0	+3.3	+83.3

*Note*: In each case, the scores are given on a scale from −100 (maximum harm) to +100 (maximum benefit) with zero being no change.

Abbreviation: GBI‐5F, 5‐factor Glasgow Benefit Inventory.

### 3.3 Sensorineural hearing loss

**FIGURE 1:**
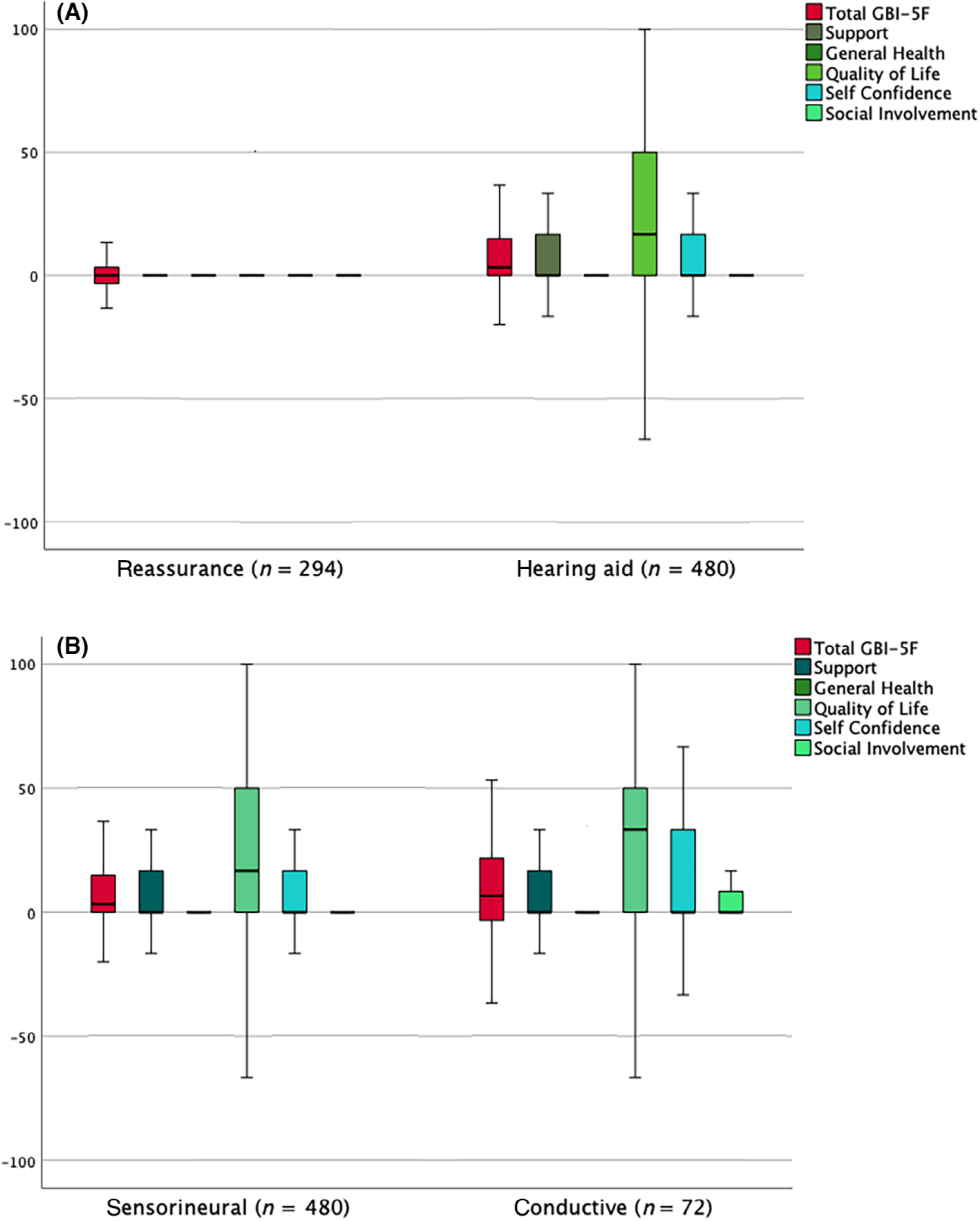
(A) Boxplot showing the *GBI‐5F* total and factor scores for patients with sensorineural hearing loss, comparing 480 patients given hearing aids with 294 given reassurance and advice only. Benefit is greater for those given hearing aids for the total score (Mann–Whitney u‐test, *p* < .001), and for the factor scores *support* (*p* = .01), *quality of life* (*p* < .001), *self‐confidence* (*p* < .001) and *social involvement* (*p* < .001) [Boxplots show the median and quartiles as the boxes, with the whiskers representing 1.5 times the interquartile range. For clarity, any outliers beyond 1.5 times the interquartile range are not shown.] (B) Boxplot showing *GBI‐5F* total and factor scores for the 480 patients given hearing aids for sensorineural hearing loss as shown in (A), but this time compared against 72 patients given hearing aids for conductive hearing loss. Benefit in the factor score *quality of life* is significantly greater in those with a conductive impairment (Mann–Whitney *U*‐test, *p* = .04). There is no significant difference in the other four factors or the overall score. GB‐5F, 5‐factor Glasgow Benefit Inventory

There were 774 patients coded as having a bilateral sensorineural hearing loss, of whom 480 received hearing aids and 294 received only reassurance and advice. Benefit is greater in those given hearing aids, with the difference between their scores and those having reassurance and advice being statistically significant for all factors except *General health* (Figure 1A).

### 3.4 Comparison of hearing aid benefit between conductive and sensorineural impairments

To make this comparison, a large cohort of those with a presumptive conductive impairment was required. A total of 72 patients were identified with a middle ear condition (28 otosclerosis, 19 inactive mucosal chronic otitis media, 17 other middle ear disorders such as adhesive otitis media and 8 previous middle ear surgery) for whom the provision of an aid was the management. Comparison of benefit **(**Figure 1B **)** showed that the *Quality of life* benefit is significantly greater in those with a conductive impairment (*n* = 72) than those with a sensorineural impairment (*n* = 480). There is no significant difference in the other four factors or the overall score.

### 3.5 Interventions for benign paroxysmal positional vertigo

**FIGURE 2:**
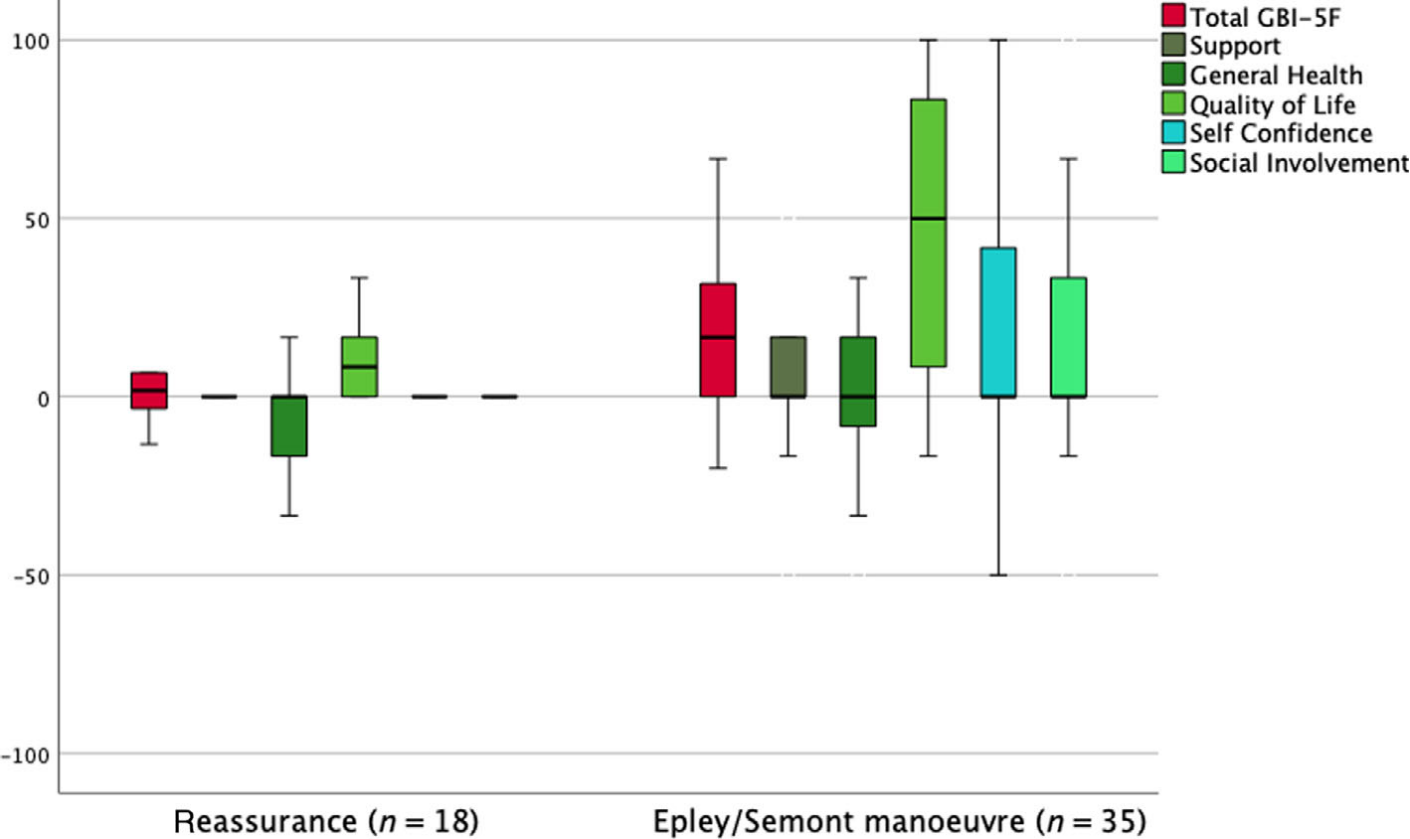
Boxplot showing *GBI‐5F* total and factor scores for the 53 patients with benign paroxysmal positional vertigo treated with reassurance and advice only (*n* = 18) versus those treated with an Epley or Semont manoeuvre (*n* = 35). There is a significant difference in the total *GBI‐5F* score (Mann–Whitney *U*‐test, *p* = .03) and the *Quality of life* factor score (*p* = .008). GBI‐5F, 5‐factor Glasgow Benefit Inventory

Of the 53 patients diagnosed with benign paroxysmal positional vertigo (BPPV), 18 patients treated with reassurance and advice only, and 35 patients receiving an Epley or Semont manoeuvre with no other intervention. There is a significant difference in the total GBI‐5F score and the *Quality of life* factor score (Figure 2), with both of these being higher in the group receiving an otolith repositioning manoeuvre.

### 3.6 Interventions for tinnitus

**FIGURE 3:**
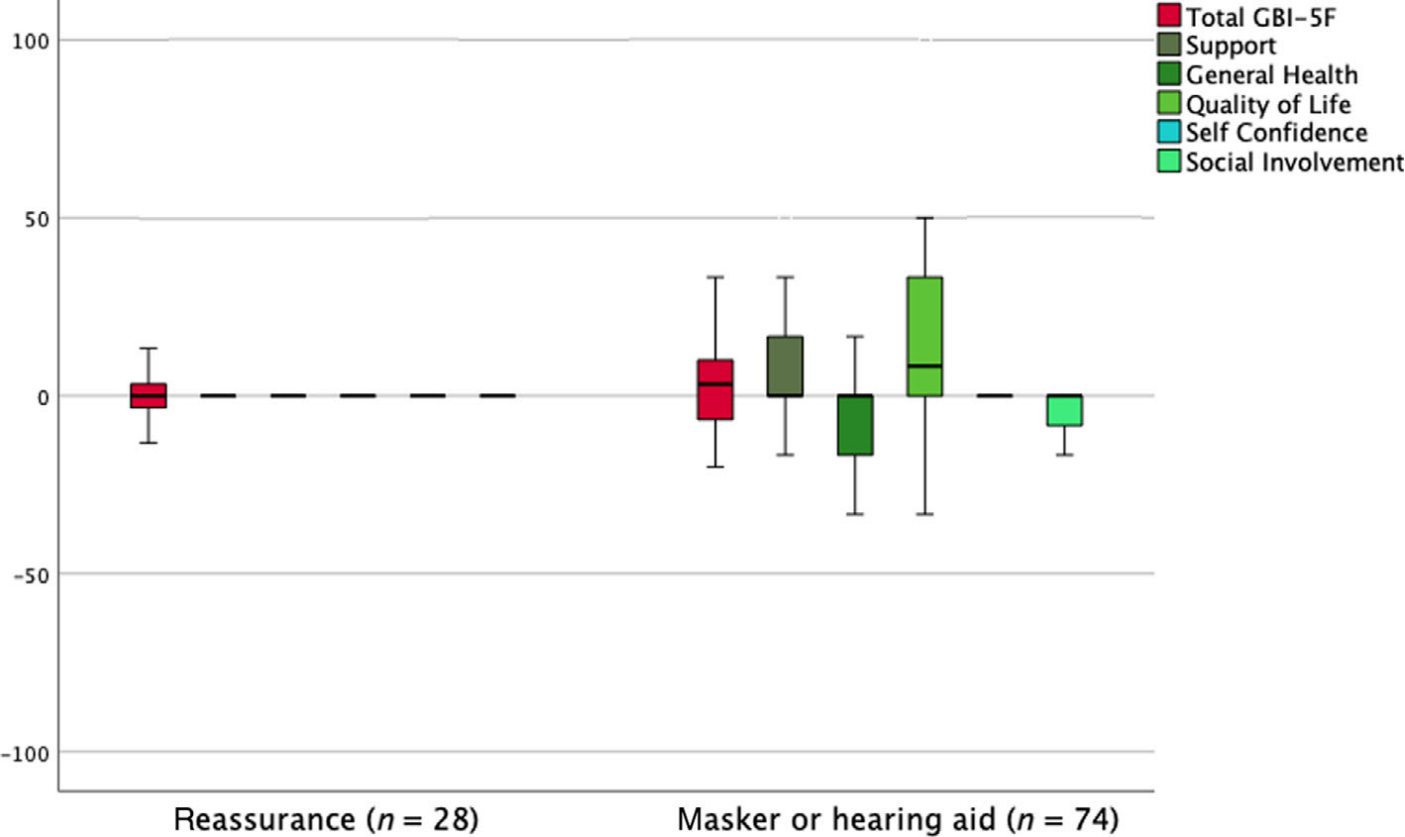
Boxplot showing *GBI‐5F* total and factor scores for 102 adults with tinnitus, comparing treatment with a tinnitus masker or hearing aid (*n* = 74) versus reassurance alone (*n* = 28). *Support* is greater in those given a hearing aid or masker (Mann–Whitney *U*‐test, *p* = .034). GBI‐5F, 5‐factor Glasgow Benefit Inventory

Of the 102 adults with tinnitus, 28 were provided with a tinnitus masker or hearing aid and 74 given reassurance alone. There was a small improvement in *Support* in those given a hearing aid or masker compared with those just given reassurance (Mann–Whitney *U*‐test, *p* = .034), but no other differences were identified (Figure 3).

### 3.7 Interventions for otitis externa

**FIGURE 4:**
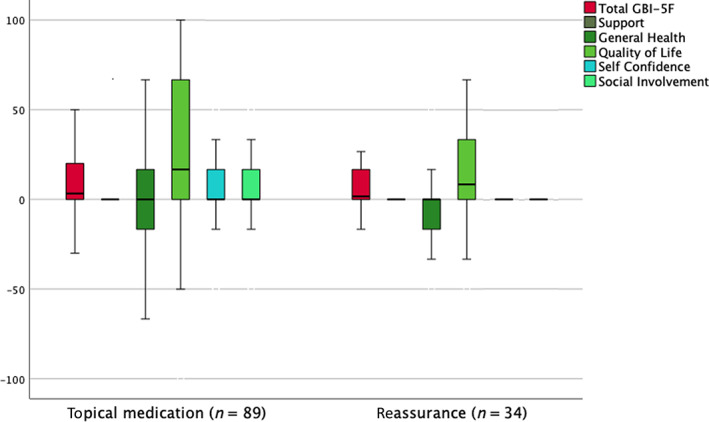
Boxplot showing *GBI‐5F* total and factor scores for the 123 patients diagnosed with otitis externa, of whom 89 were prescribed topical medications and 34 received only reassurance and advice. There is no difference in *GBI‐5F* total or factor scores between the two treatments. GBI‐5F, 5‐factor Glasgow Benefit Inventory

There were 123 patients diagnosed with otitis externa, of whom 89 were prescribed topical medications and 34 received only reassurance and advice. There is no difference in *GBI‐5F* total or factor scores between the two treatments, albeit both groups reported positive total scores and *Quality of life* factor scores **(**Figure 4 **)**.

### 3.8 Interventions for laryngo‐pharyngeal reflux

There were 195 patients with symptoms attributed to laryngo‐pharyngeal reflux of whom 176 were solely prescribed medication and 19 solely given reassurance. There is no significant difference between medication and reassurance for the total *GBI‐5F* score or any of the factor scores.

## 4 DISCUSSION

The GBI was developed to be applicable to interventions in ORL and audiology. To‐date, it has been primarily used and found valid for surgical interventions. The main objective of this article is to investigate its applicability to a wider range of ORL and audiological interventions, and specifically to do this with the recently reported version, *GBI‐5F*.

For this, we are fortunate to have a prospective national audit^4^ of 9005 adults managed in ORL and audiology departments, describing the benefit from treatment as reported by patients. Of these patients, 4543 (51%) completed a GBI questionnaire 3–6 months later and were considered by the authors of the original study to be a representative subset of all the adults attending. Of course, they are only a subset of all the patients seen during the study period and we cannot know to what extent the patients we report on here are truly representative of all patients with these particular conditions and interventions. We cannot say that the *GBI‐5F* results we report here would be typical for all patients with these conditions, but we can say that *at least some* patients with these conditions will produce scores like the ones we report. As our main intention is to show that the five‐factor scores of the GBI‐5F can be used to demonstrate the pattern of areas of benefit after different non‐surgical interventions, the question of how representative the patients are is a secondary concern. It is for future studies to report on these conditions and interventions in more detail and with reference to clinical information such as age, sex, disease severity and presenting symptoms.

In total, 80% of patients were managed without any surgery. Our data show for the first time that non‐surgical interventions can be shown to have large benefits using the *GBI‐5F*. The most striking example is that of otolith repositioning manoeuvres for BPPV, which produce benefit in the overall score and the *Quality of life* factor which are similar in magnitude to the benefits seen from the surgical treatment of conditions such as nasal polyps and tonsillitis. This would be in keeping with the dramatic and instant relief of disabling symptoms that such manoeuvres can produce.

Hearing aid provision is another non‐surgical intervention that produces significant, measurable benefit for patients with hearing loss. Those with a conductive impairment have greater *Quality of life* benefit than those with a sensorineural impairment. While we cannot control for the severity of the hearing loss in each group of patients, laboratory studies suggest that hearing aids should be more beneficial for conductive hearing impairment.^6^ More surprising, perhaps, is the lack of improvement in *Social involvement*, which is a finding that requires further investigation.

The benefit for patients with tinnitus who are given a hearing aid or a masking device is in one specific factor area (*Support*) compared with the more generalised benefit reported by patients with hearing loss (all factors except *General health*). This serves to show how five factors of the *GBI‐5F* can shed light on the details of *how* they derive benefit from specific interventions.

Of the 4543 adults with a GBI questionnaire submitted, 1373 (30%) received reassurance and advice on self‐management with no active therapy. It is important to report on these patients as they form such a large proportion of patients seen in ORL clinics. This may be because they have a condition which has settled symptomatically since referral, or because they have symptoms so minor that they do not merit active intervention. As the GBI measures a change due to an intervention, it is not surprising that the *GBI‐5F* total and factor scores were not significantly different from zero for this group. The small positive score in the *Quality of life* factor is perhaps due to the patient being reassured that there is no serious disease. It also illustrates that, in the majority of patients, the decision not to prescribe any active intervention did not lead to any harm for the patient, as any clinical deterioration over the subsequent 6 months would have produced negative scores.

For otitis externa and laryngo‐pharyngeal reflux, where there is no surgical option, medical therapies in general show no greater benefit than reassurance. This does not necessarily indicate that they are ineffective, although that could well be the case for laryngo‐pharyngeal reflux given recent evidence on the ineffectiveness of proton pump inhibitors for throat symptoms.^7^ For both these conditions, many patients have already been commenced on medication by their general practitioner prior to specialist referral: telling them to continue with medication is unlikely to produce a large reported benefit. Additionally, Q11 of the *GBI‐5F*, part of the *General Health* factor, specifically asks about medication intake, hence any intervention increasing medication will automatically worsen the *General Health* score.

We will report detailed comparisons between medication, surgery and reassurance for other conditions in a future paper, but there are some conditions where medication does lead to greater reported benefit than reassurance alone.

For individual assessment of benefit, it is important to consider what are measurable score differences. A change of one point on the answer scale (from ‘no change’ to ‘a little better’, or from ‘a little better’ to ‘much better’) for one question will produce an improvement in the overall score of +3.33, and in the relevant factor score of +16.67. The *GBI‐5F* is therefore most effective when used as tool for audit or research to assess groups of patients.

### 4.1 Strengths and weaknesses

The differences illustrated are from a large national audit completed in 2006. It is unlikely that substantially different results would be obtained on more recent data as there have been few major changes to management options for non‐malignant ORL and audiology conditions. Some might correctly argue that there have been some improvements, such as the technical advances in hearing aids. Such improvements are worth investigating and the *GBI‐5F* would be a reasonable outcome measure to do this.

Because information was not available on the severity of the ORL conditions, the benefits must be seen as a reflection of real‐world outcomes where a range of severities are managed. To show the ‘true’ magnitude of the differences requires randomised controlled trials where the severity of the disease and associated disability can be controlled as there will always be a large placebo effect when surgery, or any technological intervention such as hearing aids, is used.^8^ Where medical therapy is being investigated then it would be advisable to have a condition‐specific or symptom‐specific questionnaire in addition to the generic *GBI‐5F*.

### 4.2 Conclusion

The *GBI‐5F* is a uniquely useful tool, one which can identify differences in the magnitude of benefit from different interventions, across a wide variety of conditions, for non‐surgical as well as surgical interventions, and without the need for any pre‐intervention measurement. Therefore, it should continue to have broad application in routine audit of clinical practice and in research, especially in its revised 15‐question, 5‐factor format.

## AUTHOR CONTRIBUTIONS

George G. Browning initiated the study, supported the development of the different themes in interpretation and wrote substantial parts of the article. Haytham Kubba performed the data analyses and wrote them up, and contributed to other aspects of the article. William M. Whitmer contributed to the analysis and to drafting the article.

## FUNDING INFORMATION

WMW was supported by the Medical Research Council [grant number MR/X003620/1] and the Chief Scientist Office of the Scottish Government.

## CONFLICT OF INTEREST

The author declares that there is no conflict of interest that could be perceived as prejudicing the impartiality of the research reported.

### PEER REVIEW

The peer review history for this article is available at https://publons.com/publon/10.1111/coa.13992.

## ETHICS STATMENT

This study was performed on pre‐existing data, originally obtained for the Scottish ENT Outcomes Study which was approved by the Scottish Multi‐Centre Research Ethics Committee.

## Data Availability

The data that support the findings of this study are available from the corresponding author upon reasonable request.
